# Role of Bone Marrow-Derived Fibroblasts in Renal Fibrosis

**DOI:** 10.3389/fphys.2016.00061

**Published:** 2016-02-25

**Authors:** Jingyin Yan, Zhengmao Zhang, Li Jia, Yanlin Wang

**Affiliations:** ^1^Selzman Institute for Kidney Health and Section of Nephrology, Department of Medicine, Baylor College of MedicineHouston, TX, USA; ^2^Renal Section, Michael E. DeBakey Veterans Affairs Medical CenterHouston, TX, USA

**Keywords:** chemokine, cytokine, circulating fibroblast precursors, fibroblasts, renal fibrosis, extracellular matrix, chronic kidney disease, monocyte-to-fibroblast transition

## Abstract

Renal fibrosis represents a common pathway leading to progression of chronic kidney disease. Renal interstitial fibrosis is characterized by extensive fibroblast activation and excessive production and deposition of extracellular matrix (ECM), which leads to progressive loss of kidney function. There is no effective therapy available clinically to halt or even reverse renal fibrosis. Although activated fibroblasts/myofibroblasts are responsible for the excessive production and deposition of ECM, their origin remains controversial. Recent evidence suggests that bone marrow-derived fibroblast precursors contribute significantly to the pathogenesis of renal fibrosis. Understanding the molecular signaling mechanisms underlying the recruitment and activation of the bone marrow-derived fibroblast precursors will lead to novel therapy for the treatment of chronic kidney disease. In this review, we summarize recent advances in our understanding of the recruitment and activation of bone marrow-derived fibroblast precursors in the kidney and the development of renal fibrosis and highlights new insights that may lead to novel therapies to prevent or reverse the development of renal fibrosis.

## Introduction

Chronic kidney disease (CKD) is a global public health problem. Worldwide, over 1 million people die from CKD yearly. It is estimated that more than 20 million Americans have CKD and more than 450,000 Americans suffer from end-stage renal disease (ESRD) requiring renal replacement therapy. CKD has become the eighth leading cause of death in the United States. Regardless of various etiologies, a common pathological feature of CKD is renal fibrosis, which is characterized by accumulation of fibroblasts/myofibroblasts with increased production and deposition of extracellular matrix (ECM) including collagen type I, III, IV, fibronectin, vimentin, and proteoglycans (Zeisberg and Neilson, 2010; Conway and Hughes, 2012; Farris and Colvin, 2012). It is generally agreed that activated fibroblasts termed myofibroblasts are the principle cells responsible for the increase ECM production and deposition, the origin of these matrix-producing cells remains debatable. They were traditionally thought to arise from resident interstitial fibroblasts (Qi et al., 2006; Picard et al., 2008). Recent evidence indicate that they may originate from epithelial/endothelial-to-mesenchymal transition (Iwano et al., 2002; Zeisberg and Kalluri, 2004; Zeisberg et al., 2008; Liu, 2010), pericytes (Lin et al., 2008), and bone marrow-derived fibroblast precursors termed fibrocytes (Grimm et al., 2001; Sakai et al., 2006; Broekema et al., 2007; Niedermeier et al., 2009; Chen et al., 2011).

In response to kidney injury, multiple cell types in the circulation are recruited to the site of injury to participate in a wound healing response. A dysregulated wound healing process causes fibrosis, where ECM and fibroblasts replace normal renal parenchyma and lead to kidney dysfunction. Therefore, investigations of underlying mechanisms and identification of novel therapeutic targets of renal fibrosis will have huge impact on patient survival, global health, and economic burden. Fibrosis is a complex and progressive pathological process involving infiltration of mononuclear cells including bone marrow-derived fibroblast precursors (fibrocytes), macrophages, and immune cells, which suggest that interaction and communication among these cell types regulates the development of fibrotic disorders (Kisseleva and Brenner, 2008; Wynn, 2008). Because activated fibroblasts are the principal effector cells that mediate ECM production in the fibrotic kidney disease, their activation is regarded as an important event in the pathogenesis of renal fibrosis (Eddy, 2005; Neilson, 2006). However, the origin of the activated fibroblasts has been controversial. They are traditionally thought to arise from resident fibroblasts within the kidney. Recent studies have shown that the activated fibroblasts may originate from bone marrow-derived fibroblast precursors (Grimm et al., 2001; Iwano et al., 2002; Sakai et al., 2006; Broekema et al., 2007; Chen et al., 2011). In a clinical study of mismatched kidney transplantation in humans, the proportion of host-derived smooth muscle actin (SMA)-positive cells is ~30% in allografts undergoing chronic rejection compared with 10% in those without rejection (Grimm et al., 2001). In rodent models of renal fibrosis, we and others have shown that bone marrow-derived fibroblasts migrate into the kidney in response to injury (Iwano et al., 2002; Roufosse et al., 2006; Broekema et al., 2007; Chen et al., 2011; Xia et al., 2014b). For example, one study using bone marrow transplantation of transgenic mice that express enhanced green fluorescence protein (GFP) under the control of the fibroblast specific protein 1 (FSP1) promoter has demonstrated that 15% of bone marrow-derived fibroblasts are present in the kidney 10 days after obstructive injury (Iwano et al., 2002). Using bone marrow transplantation of transgenic rats that express human placental alkaline phosphatase, Broekema et al. have shown that more than 30% α-SMA positive myofibroblasts are derived from bone marrow 7 days after ischemia-reperfusion injury (Broekema et al., 2007). Using chimeric mice transplanted with GFP-expressing bone marrow cells, Li et al have reported that more than 30% of renal α-SMA+ myofibroblasts are derived from the bone marrow in a mouse adriamycin-induced fibrosis model (Li et al., 2007). Recently, Lebleu et al. have demonstrated that 35% α-SMA positive myofibroblasts are derived from bone marrow 10 days after obstructive injury using bone marrow transplantation of transgenic mice express red fluorescence protein (RFP) driven by α-SMA (Lebleu et al., 2013). Using bone marrow transplantation of transgenic mice that express GFP driven by collagen 1A1 promoter, we have shown that bone marrow-derived hematopoietic fibroblasts migrate into the kidney, proliferate, and differentiate into α-SMA^+^ myofibroblasts (Xia et al., 2014b). In contrast, one study reported that bone marrow-derived GFP^+^ fibroblasts contribute to a minor fraction of myofibroblasts (Lin et al., 2008). Another study reported that bone marrow-derived cells do not contribute significantly to collagen synthesis using bone marrow transplantation of transgenic mice that express luciferase and β-galactosidase driven by collagen 1A2 promoter (Roufosse et al., 2006). One potential pitfall of bone marrow transplantation is the bone marrow engraftment rate. Furthermore, detection of GFP, luciferase, or β-galactosidase in tissue can be technically demanding. Therefore, it is difficult to interpret negative data obtained with these reporter mice. To objectively quantify the number of hematopoietic fibroblasts in the kidney, we stained freshly-isolated kidney cells with CD45, a hematopoietic cell marker, and collagen I, a mesenchymal cell marker, and examined with flow cytometry. The results have shown that CD45^+^ and collagen I^+^ cells constituted 45% of total collagen I^+^ cells in the kidney 7 days after obstructive injury (Xia et al., 2014b). Using lineage tracing and adoptive transfer, Wang et al. have recently reported that monocytes/macrophages from bone marrow can transform into myofibroblasts via macrophage-to-myofibroblast transition (Wang et al., 2015). Therefore, compelling evidence indicate that bone marrow-derived fibroblasts contribute significantly to the pathogenesis of renal fibrosis and suggest targeting bone marrow-derived fibroblasts may represent a novel therapeutic strategy for the treatment of fibrotic kidney disease and possible fibrotic disorders of other organs.

## Recruitment of bone marrow-derived fibroblast precursors

The recruitment of circulating cells into sites of injury is mediated by locally produced chemokines. Chemokines are classified based on the relative position of cysteine residues near the NH2 terminus into four major families: CC, CXC, C, and CX3C (Rollins, 1997; Mackay, 2001). Recent studies have shown that chemokines and their receptors play an important role in the recruitment of bone marrow-derived fibroblast precursors into the kidney in response to injury. CCL21/CCR7, CXCL16/CXCR6, CCR2 are involved in recruiting circulating fibroblast precursors into the kidney (Table 1) (Sakai et al., 2006; Chen et al., 2011; Reich et al., 2013; Xia et al., 2013a,b, 2014a,b).

**Table 1:** **Chemokines and their receptors in the recruiment of bone marrow-derived fibroblast precusors or fibrocytes**.

**Chemokines/Receptors**	**Methods**	**Models**	**Effect on fibrosis**	**References**
CCL21/CCR7	Anti-CCL21 antibody CCR7 knockout mice	UUO	40–50% reduction	Sakai et al., 2006
CXCL16	CXCL16 knockout mice	UUO	40–50% reduction	Chen et al., 2011
CCR2	CCR2 knockout mice, depletion of fibrocytes, BMT	UUO	20–30% reduction	Reich et al., 2013
CCR2	CCR2 knockout mice	UUO	30–40% reduction	Xia et al., 2013a
CXCL16	CXCL16 knockout mice	Angiotensin II infusion	40–50% reduction	Xia et al., 2013b
CXCR6	CXCR6 knockout mice, BMT	Angiotensin II infusion	40–50% reduction	Xia et al., 2014a
CXCR6	CXCR6 knockout mice, BMT	UUO, IRI	40–50% reduction	Xia et al., 2014b

BMT, bone marrow transplantation; IRI, ischemia-reperfusion injury; UUO, unilateral ureteral obstruction.

The chemokine CXCL16 is a recently discovered cytokine belonging to the CXC chemokine family (Matloubian et al., 2000; Wilbanks et al., 2001). It was originally described as a scavenger receptor for phosphatidylserine and oxidized low-density lipoprotein (SR-PSOX; Shimaoka et al., 2000). There are two forms of CXCL16. The transmembrane form is a type I transmembrane glycoprotein consisting of an extracellular N-terminal chemokine domain, glycosylated mucin-like stalk, transmembrane-spanning region, and a short cytoplasmic domain, with a YXPV motif that is a potential tyrosine-phosphorylation and SH2-protein-binding site (Izquierdo et al., 2014). The transmembrane form functions as an adhesion molecule for CXCR6 expressing cells and scavenger receptor for oxidized low-density lipoprotein (Shimaoka et al., 2000, 2004). The soluble form generated by its cleavage at the cell surface functions as a chemoattractant to recruit circulating cells (Gough et al., 2004). CXCL16 is induced in kidney tubular epithelial cells *in vivo* in a murine model of renal fibrosis induced by obstructive injury (Okamura et al., 2007; Chen et al., 2011). Tumor necrosis factor (TNF)-α and interferon (IFN)-γ upregulate CXCL16 expression in tubular epithelial cells *in vitro* (Xia et al., 2013a). In addition, the TNF superfamily cytokine TNF-like weak inducer of apoptosis (TWEAK) increases CXCL16 expression in kidney tubular epithelial cells *in vitro* and *in vivo* (Izquierdo et al., 2012). Interestingly, angiotensin II, a key promoter of kidney injury, also enhances CXCL16 expression in tubular epithelial cells via activation of NF-kB, a master regulator of inflammation (Xia et al., 2013b). We have recently studied the functional role of CXCL16 in the pathogenesis of renal fibrosis in a murine model of obstructive nephropathy using CXCL16 knockout mice. Our results have shown that bone marrow-derived fibroblast precursor infiltration, myofibroblast activation, and ECM protein deposition are reduced in the obstructed kidneys of CXCL16-knockout mice (Chen et al., 2011). These data indicate that CXCL16 promotes renal fibrosis by recruiting bone marrow-derived fibroblast precursors. More recently, we have examined the functional role of CXCL16 in angiotensin II-induced renal injury and fibrosis. Our results have demonstrated that genetic deletion of CXCL16 protects the kidney from angiotensin II infusion-induced renal dysfunction, inhibits renal fibrosis, reduces proteinuria, suppresses bone marrow-derived fibroblast accumulation, myofibroblast formation, macrophage, and T cell infiltration and pro-inflammatory cytokine expression without affecting blood pressure at baseline or in response to angiotensin II infusion (Xia et al., 2013b). In support of clinical relevance of these observations in experimental animal models, clinical studies have shown that circulating CXCL16 is elevated in patients with CKD and diabetic nephropathy and high levels of CXCL16 are associated with CKD progression and development of proteinuria (Lin et al., 2011; Zhao et al., 2014).

CXCR6 is the receptor for CXCL16. CXCR6 was first cloned as an orphan receptor by three independent groups and was termed STRL33, BONZO, or TYMSTR (Alkhatib et al., 1997; Deng et al., 1997; Loetscher et al., 1997). We have recently shown that both circulating fibroblast precursors and bone marrow-derived fibroblasts in the kidney express CXCR6 (Chen et al., 2011; Xia et al., 2014b). Genetic disruption of CXCR6 reduces the recruitment of bone marrow-derived fibroblast precursors into the kidney and the development of renal fibrosis induced by ureteral obstruction, ischemia-reperfusion, and angiotensin II infusion (Xia et al., 2014a,b).

In these studies, we have observed that genetic deficiency of CXCL16 or CXCR6 does not completely block bone marrow-derived fibroblast precursor infiltration into the kidney and renal fibrosis development, suggesting that other chemokine/receptor pairs may be involved in the process of recruiting bone marrow-derived fibroblast precursors into the kidney. Consistent with this notion, CCR2 and CCL21/CCR7 have been reported to play a role in the recruitment of bone marrow-derived fibroblast precursors into the kidney and the development of renal fibrosis (Sakai et al., 2006; Reich et al., 2013; Xia et al., 2013a). Interestingly, the expression of CXCL16 in the kidney is reduced in CCR2 knockout mice in response to obstructive injury, suggesting the interaction of two distinct chemokine systems modulates renal tubular epithelial cell-initiated fibrosis (Xia et al., 2013a).

## Activation of bone marrow-derived fibroblast precursors

### TGF-β1/Smad3

The activation of bone marrow derived fibroblast precursors plays a crucial role in the pathogenesis of renal fibrosis (Yang et al., 2013; Chen et al., 2014). Myofibroblasts are a population of smooth muscle-like fibroblasts that express α-smooth muscle actin (α-SMA; Powell et al., 1999). The activation of myofibroblasts is generally considered a main event in the pathogenesis of renal fibrosis (Nath, 1992; Eddy, 2013). Furthermore, experimental and clinical studies have shown that the number of interstitial myofibroblasts is associated closely with the severity of tubulointerstitial fibrosis and the rapidity of kidney disease progression (Zhang et al., 1995; Essawy et al., 1997; Roberts et al., 1997). The activation of bone marrow-derived fibroblast precursors are regulated by locally produced cytokines (Yang et al., 2013; Chen et al., 2014). TGF-β1 is a key profibrotic cytokine that play an important role in the pathogenesis of renal fibrosis through activation of a cascade of intracellular signaling pathways (Border et al., 1990; Border and Noble, 1994; Böttinger and Bitzer, 2002; Lan, 2011). Evidence suggests that activation of the canonical Smad signaling cascade plays an important role in stimulating ECM protein expression and tissue fibrosis (Verrecchia et al., 2001; Zhao et al., 2002; Sato et al., 2003; Latella et al., 2009; Huang et al., 2010; Meng et al., 2015). We have recently examined the functional role of Smad3 in the activation of bone marrow-derived fibroblast precursors *in vitro* and *in vivo* (Chen et al., 2014). In cultured monocytes, TGF-β1 activates Smad3. Smad3 deficient monocytes express less amount ECM proteins at baseline and Smad3 deficiency completely abolished TGF-β1-induced expression of α-SMA and extracellular matrix proteins in cultured monocytes *in vitro*. Smad3-knockout mice accumulate significantly fewer bone marrow-derived fibroblasts in the kidney after obstructive injury, exhibit less myofibroblast activation, and express less α-SMA in the obstructed kidney. Furthermore, genetic deletion of Smad3 reduces total collagen deposition and suppresses expression of extracellular matrix proteins. Additionally, wild-type mice engrafted with Smad3^−∕−^ bone marrow cells displayed fewer bone marrow-derived fibroblasts in the kidney with obstructive injury and showed less severe renal fibrosis compared with wild-type mice engrafted with Smad3^+∕+^ bone marrow cells. These results indicate that Smad3 of bone marrow-derived cells plays an important role in bone marrow-derived fibroblast activation. However, Samd3 deficiency does not completely abolish bone marrow-derived fibroblast activation, collagen deposition, and ECM protein expression *in vivo*. These results suggest that other factors may be involved in bone marrow-derived fibroblast activation.

### JAK3/STAT6

The activation of bone marrow-derived fibroblasts is modulated by inflammatory cells in the microenvironment. T cells plays an important role in the pathogenesis of renal fibrosis (Tapmeier et al., 2010), which have been reported to regulate bone marrow-derived fibrocyte activation (Niedermeier et al., 2009). Naïve CD4^+^ T cells can differentiate into two major distinct phenotypes, Th1 and Th2 cells, which are characterized by specific cytokine expression patterns (Wynn, 2004). Th2 cells produce Th2 cytokines such as IL-4 and IL-13, which induce alternative activation of macrophage and promotes monocyte-to-fibroblast transition, Th1 cells produces Th1 cytokines such as IFN-γ and IL-12, which promote classical activation of macrophages and inhibit fibrocyte differentiation (Wynn, 2004; Shao et al., 2008). However, the molecular signaling mechanisms by which Th2 cytokines promote bone marrow-derived fibroblast activation are not known. We have found that JAK3/STAT6 signaling pathway is activated during the development of renal fibrosis and plays an important role in bone marrow-derived fibroblast activation, extracellular matrix protein production, and interstitial fibrosis development (Yan et al., 2015). Specifically, our results have shown that Th2 cytokine—IL-4 or IL-13 induces STAT6 activation and stimulates bone marrow monocytes to express ECM proteins and α-smooth muscle actin (α-SMA). CP690550, a JAK3-specific inhibitor, or STAT6 deficiency inhibits IL-4/IL-13-indcued STAT6 activation and expression of ECM proteins and α-SMA in bone marrow monocytes *in vitro*. Furthermore, CP690550 treatment or STAT6 deficiency inhibits bone marrow-derived fibroblast activation and ECM protein production in the kidney in response to obstructive nephropathy. To further confirm the role of bone marrow STAT6 signaling in myeloid fibroblast activation, we performed bone marrow chimeric experiments. Wild-type mice transplanted with STAT6 null bone marrow cells exhibit fewer bone marrow-derived fibroblasts and develop a lesser degree of renal fibrosis. These results suggest that inhibition of JAK3/STAT6 signaling may serve as a novel therapeutic target for fibrotic kidney disease.

### Adiponectin/AMPK

Adiponectin is a multifunctional cytokine and an important regulator of lipid and carbohydrate metabolism. Emerging evidence suggests that circulating adiponectin levels are elevated in patients with CKD, and a high level of adiponectin is linked to increased cardiovascular mortality (Zoccali et al., 2003; Shimotomai et al., 2005; Iwashima et al., 2006; Zoccali and Mallamaci, 2011; Mills et al., 2013). We have discovered that adiponectin is induced following ischemia-reperfusion and obstructive injury (Yang et al., 2013). Genetic deletion of adiponectin inhibits bone marrow-derived fibroblast accumulation and myofibroblast activation. Furthermore, adiponectin deficiency also reduces the expression of profibrotic chemokines and cytokines and the production of ECM protein in the kidneys following obstructive injury or ischemia-reperfusion. These results indicate that adiponectin plays a significant role in the activation and maturation of bone marrow-derived fibroblasts and the development of renal fibrosis. Mechanistically, adiponectin stimulated α-SMA and extracellular matrix protein expression in bone marrow-derived monocytes via activation of adenosine monophosphate-activated protein kinase (AMPK). AMPK inhibition with a pharmacological inhibitor (compound C) or dominant negative AMPK-α1 attenuated adiponectin-induced expression of α-SMA and extracellular matrix proteins. Furthermore, AMPK activation with 5-aminoimidazole-4-carboxamide-riboside (AICAR), a cell permeable adenosine analog (Corton et al., 1995), resulted in increased expression of α-SMA and extracellular matrix proteins. These results indicate that adiponectin is a critical regulator of monocyte-to-fibroblast transition and renal fibrosis. Therefore, inhibition of adiponectin/AMPK signaling may represent a novel therapeutic target for fibrotic kidney disease.

It is generally thought that macrophages do not produce ECM proteins. These cells promote fibrosis indirectly by producing profibrotic cytokines that activate fibroblasts (Wynn and Barron, 2010). Recently, a model of two major macrophage classes has been proposed. Classically activated macrophages exhibit a Th1-like phenotype and promote inflammation in response to Th1 cytokines; while alternatively activated macrophages or M2 macrophages display a Th2-like phenotype and stimulate ECM production in response to Th2 cytokines (Gordon and Martinez, 2010). M2 macrophages are characterized by expressing MHC class II, mannose receptor (CD206), Fizz1/Relm-α, and arginase. Alternatively activated macrophages are implicated in the fibrogenesis of other organs (Gordon and Martinez, 2010). The functional role of macrophages has been intensively investigated using a variety of depletion/blocking strategy in the UUO model of renal fibrosis. Kitagawa et al. reported that genetic deletion of CCR2 or treatment with CCR2 inhibitors reduces the infiltration of F4/80-positive macrophages into the kidney and attenuates the development of renal fibrosis (Kitagawa et al., 2004). Depletion of macrophages using CD11b-DTR mice reduces myofibroblast accumulation and the degree of renal fibrosis through regulation of galectin-3 expression in the kidney (Henderson et al., 2008). Interestingly, Galectin-3 has been shown to promote M2 macrophage polarization (MacKinnon et al., 2008). Selectively depletion of F4/80-positive cells with liposome clodronate attenuates the development of renal fibrosis, which is associate with a reduction of TNF-α and TGF-β gene expression (Kitamoto et al., 2009). However, not all macrophage depletion/inhibition strategies result in a reduction of renal fibrosis in the UUO model. Depletion of leukocyte with cyclophosphamide increases the degree of renal fibrosis, which is attenuated by adoptive transfer of macrophages (Nishida et al., 2005). Furthermore, inhibition of macrophage colony-stimulating factor receptor kinase reduces macrophage accumulation and tubular apoptosis without affecting the development of renal fibrosis (Ma et al., 2009). These studies may reflect the functional heterogeneity of the macrophage subsets. We have recently demonstrated for the first time that alternatively activated macrophages produce procollagen I, suggesting a link between M2 macrophage polarization and monocyte-to-fibroblast transition (Yang et al., 2013). Consistent with this novel concept, we have shown that adiponectin deficiency suppresses M2 macrophage polarization and inhibits the number of collagen-expressing M2 macrophages in the injured kidneys (Yang et al., 2013). Therefore, macrophages can participate in the pathogenesis of renal fibrosis via direct and indirect mechanisms (Meng et al., 2014; Nikolic-Paterson et al., 2014).

### Renin angiotensin system

Recent studies have shown that renin angiotensin system plays an important role in the development of fibrotic kidney disease (Mezzano et al., 2001). Inhibition of renin angiotensin system with ACE inhibitors or angiotensin type 1 blockers attenuates experimental renal fibrosis development in animals and retards CKD progression in humans (Ishidoya et al., 1995; Brenner et al., 2001). Sakai et al. have examined the role of renin angiotensin system in the regulation of fibrocytes in murine models of renal fibrosis (Sakai et al., 2008). Their results have shown that inhibition of angiotensin II type 1 receptor (AT1R) with valsantan reduces the number of fibrocytes in the kidney and bone marrow and inhibits the development of renal fibrosis. In contrast, angiotensin II type 2 receptor (AT2R) knockout mice exhibit increased number of fibrocytes in the kidney and bone marrow and develop more severe renal fibrosis. Furthermore, inhibition of AT1R with valsantan decreases angiotensin II-induced collage type I and TGF-β1 expression while inhibition of AT2R increases angiotensin II-induced collage type I and TGF-β1 expression in cultured fibrocytes. This study indicates that angiotensin II receptors play opposite role in the development and activation of fibrocytes. Further studies are needed to dissect the downstream signaling mechanisms underlying angiotensin II-induced fibrocyte activation.

## Conclusion

**Figure 1:**
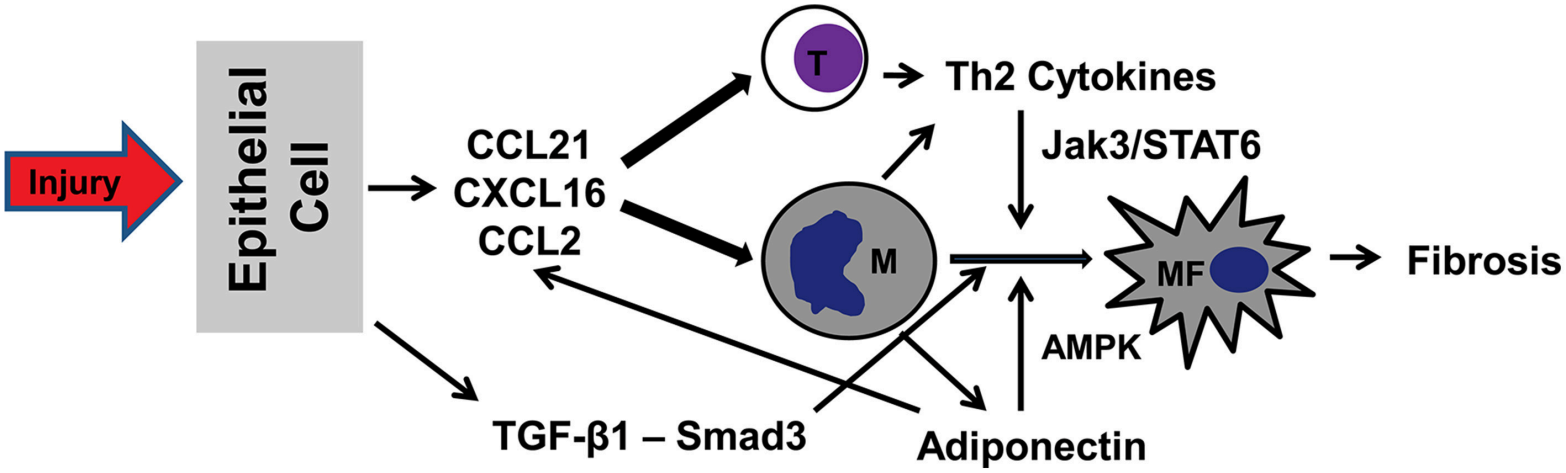
**A proposed model of signaling mechanisms underlying recruitment and activation of bone marrow-derived fibroblasts in renal fibrosis**. In response to injury (urinary tract obstruction, ischemia-reperfusion, hypertension), tubular epithelial cells produce chemokines CCL21/CXCL16/CCL2 and cytokine TGF-β1. Chemokines act concertedly to recruit bone marrow-derived cells (T cells, monocytes, and fibrocytes) via interaction with their respective receptors. TGF-β1 activates Smad3 to stimulate monocyte-to-fibroblast transition. Th2 cells produce Th2 cytokines, which activate JAK3/STAT6 signaling pathway to promote monocyte-to-fibroblast transition. Finally, adiponectin produced by infiltrating inflammatory cells regulates chemokine and cytokine production and stimulates monocyte-to-fibroblast transition through activation of AMPK. T, T cells; M, Monocytes; MF, Myeloid fibroblasts.

Recent studies have demonstrated that bone marrow derived fibroblast precursors contribute significantly to the pathogenesis of renal fibrosis. Recruitment and activation of bone marrow-derived fibroblasts are mediated through the interaction between chemokines/cytokines and their receptors (Figure 1). Therefore, targeting the signaling machinery of these chemokines/cytokines could represent novel therapeutic strategy for the treatment of renal fibrosis and possible fibrotic disorders of other organs.

## Future perspective

Although chemokines are involved in recruiting bone marrow-derived fibroblasts into the kidney in response to injury, the molecular signaling mechanisms underlying chemokines-induced bone marrow-derived fibroblast recruitment remains to be defined. Ras proteins are members of a family of small GTPase that control signaling pathways involved in cell migration, proliferation, differentiation, and survival (Rodríguez-Pena et al., 2005). There are three Ras proteins, H-Ras, K-Ras, and N-Ras. These Ras proteins are ubiquitously expressed including the kidney. Recent studies have shown that Ras proteins are involved in the development of renal fibrosis. Grande et al. have reported that genetic gelation of H-Ras inhibits myofibroblast activation and renal fibrosis development following ureteral obstruction in mice (Grande et al., 2010). Future studies are needed to define the role of Ras signaling in the regulation of fibrocyte migration, proliferation, and differentiation.

## Author contributions

JY drafted the manuscript. ZZ, LJ, and YW reviewed and edited the manuscript.

### Conflict of interest statement

The authors declare that the research was conducted in the absence of any commercial or financial relationships that could be construed as a potential conflict of interest.
